# Assessing the potential for application of machine learning in predicting weather-sensitive waterborne diseases in selected districts of Tanzania

**DOI:** 10.3389/frai.2025.1597727

**Published:** 2025-06-04

**Authors:** Neema Nicodemus Lyimo, Kadeghe Goodluck Fue, Silvia Francis Materu, Ndimile Charles Kilatu, Joseph Philipo Telemala

**Affiliations:** ^1^Department of Informatics and Information Technology, Sokoine University of Agriculture, Morogoro, Tanzania; ^2^Department of Engineering Sciences and Technology, Sokoine University of Agriculture, Morogoro, Tanzania; ^3^Department of Biosciences, Sokoine University of Agriculture, Morogoro, Tanzania; ^4^Department of Health, Morogoro Municipal Council, Morogoro, Tanzania

**Keywords:** climate-sensitive, artificial intelligence, Tanzania, predictive ML, waterborne diseases, developing countries

## Abstract

**Introduction:**

This study evaluates the potential of machine learning (ML) to predict and manage weather-sensitive waterborne diseases (WSWDs) in selected Tanzanian districts, focusing on environmental health officers' (EHOs) knowledge and perceptions. It explores EHOs' familiarity with information and communication technology (ICT) and artificial intelligence (AI)/ML, alongside challenges and opportunities for integrating AI-driven public health solutions.

**Methods:**

A census-style survey was conducted among EHOs in three district councils. A structured questionnaire, piloted in one district, was administered to 76 EHOs, achieving a 66% response rate. Data were analyzed using descriptive and inferential statistics to assess knowledge levels, perceptions, and gender-related differences.

**Results:**

Most EHOs were moderately familiar with ICT; however, only 54% had prior exposure to AI/ML concepts, and 64% reported limited AI familiarity. Among the variables examined, only prior exposure to AI/ML concepts and self-reported familiarity with AI demonstrated statistically significant associations with gender. Despite this, the majority recognized AI/ML's potential to improve disease prediction accuracy. Key barriers to ML adoption include inadequate technical infrastructure, data quality issues, and a shortage of expertise. Opportunities identified included utilizing historical disease data, integrating AI with meteorological information, and using satellite imagery for surveillance.

**Discussion:**

The study highlights frontline health workers' perceived barriers to ML adoption and suggests that gender influences awareness and engagement with AI and ML technologies. Strengthening technical capacity, improving data quality, and fostering cross-sector collaboration are critical for successful AI/ML integration. These insights offer a roadmap for resilience to WSWDs in developing countries like Tanzania through data-driven technologies.

## 1 Introduction

The intersection of climate change and public health presents significant challenges, particularly in low-income countries like Tanzania, where water insecurity and waterborne diseases are prevalent (Mutono et al., 2021). Changes in rainfall patterns, rising temperatures, and extreme weather events driven by climate change exacerbate water quality issues, increasing the incidence of diseases such as cholera, diarrhea, and typhoid. In sub-Saharan Africa (SSA), including Tanzania, frequent extreme hydrological events—such as floods and droughts—result in water contamination and shortages, further compromising public health (Mutono et al., 2021; World-Bank, 2024). During flooding, runoff from pit latrines contaminates open-dug wells and rivers, heightening the risk of disease transmission (Mtwangi Limbumba et al., 2019; Mguni et al., 2016; Pantaleo et al., 2018). Conversely, droughts reduce access to clean water, creating hygiene challenges that foster environments conducive to disease outbreaks (Hunter et al., 2010; Howard et al., 2016).

The urban population in Tanzania is particularly vulnerable to these climate-sensitive diseases due to rapid urbanization and inadequate infrastructure development (Mshida et al., 2020). According to a report from WHO/UNICEF Joint Monitoring Programme for Water Supply, Sanitation, and Hygiene (2018/2019), only 47% of the urban population in Tanzania has access to basic sanitation, and merely 23.5% has access to basic hygiene facilities (Mshida et al., 2020). This lack of access to safe water increases the risk of diseases such as amoebiasis, typhoid fever, and dysentery among the population, particularly affecting children under 5 years (WHO, 2024). Nearly 1.7 billion cases of childhood diarrheal disease occur globally each year, ranking as the third leading cause of death among children aged 1–59 months (Adedokun and Yaya, 2020). In Tanzania, inadequate water, sanitation, and hygiene (WASH) services contribute to over 31,000 annual deaths and result in economic losses exceeding USD 2.4 billion due to increased medical expenses and reduced productivity (World-Bank, 2024). Studies have shown a direct correlation between rising temperatures and increased disease risk. For instance, for every 1°C increase in temperature, the likelihood of cholera outbreaks can rise by 15%–29% (Trærup et al., 2011). Additionally, erratic rainfall patterns elevate the risk of water contamination, further endangering public health (Mboera et al., 2011). Children from poorer households in SSA, particularly those living in areas with unimproved sanitation, face a higher risk of contracting waterborne diseases (Flückiger and Ludwig, 2022; Demissie et al., 2021; Adedokun and Yaya, 2020).

In response to these challenges, technological advancements, particularly in artificial intelligence (AI) and machine learning (ML), offer promising solutions for predicting and managing outbreaks of waterborne diseases. Research highlights various applications of AI in health sectors of low- and middle-income countries, including epidemiological surveillance and disease prediction (Adigwe et al., 2024). ML algorithms have shown remarkable potential in analyzing large datasets to identify patterns that can forecast disease outbreaks, often detecting anomalies that traditional methods might overlook. Recent studies highlight the effectiveness of ML models, such as Random Forest and Support Vector Machines (SVMs), in predicting disease cases based on historical patient data, achieving up to 77% accuracy for typhoid prediction when key input features like age and medical history are incorporated (Hussain et al., 2023; Flückiger and Ludwig, 2022). Additionally, IoT-based systems for real-time water quality monitoring have demonstrated their utility in early detection of potential health risks (Nemade et al., 2024). ML techniques, including deep learning models such as Long Short-Term Memory Networks (LSTMs) (Hochreiter and Schmidhuber, 1997) and Convolutional Neural Networks (CNNs) (LeCun et al., 1998), have been widely applied in healthcare research for developing predictive and diagnostic models. For example, Jia et al. (2019) used LSTM models to predict typhoid outbreaks, while Guo et al. (2020) employed SVM models for hepatitis prediction.

Familiarity with AI/ML among EHOs is a critical factor in the practical implementation of these technologies for disease monitoring and prediction. In many low-resource settings, including regions in South Asia and sub-Saharan Africa, studies have emphasized the importance of local health professionals' digital literacy in influencing the success of AI-driven interventions (Rajkomar et al., 2019; Topol, 2019). For instance, a review by Rasheed et al. (2020) found that AI approaches significantly supported frontline workers and decision-makers during the COVID-19 pandemic by enhancing real-time diagnostics, optimizing resource allocation, and improving situational awareness, thereby reducing the burden on healthcare systems and improving response effectiveness. A study by Chettri et al. (2025) highlights that in the Indian healthcare sector, the integration of trustworthy AI systems faces challenges due to ethical and regulatory constraints, emphasizing the need for capacity building among healthcare workers to bridge this gap. Similarly, a scoping review by Botha et al. (2024) underscores the perceived threats posed by AI tools in healthcare, such as unpredictable errors and data privacy concerns, which can be mitigated through proper training and involvement of healthcare professionals in the deployment process. These insights underscore that the familiarity and acceptance of AI/ML among EHOs not only facilitates smooth adoption but also enhances the relevance and contextual accuracy of such tools in mitigating climate-sensitive health risks.

Despite the potential of these technologies, their application in Tanzania remains limited, with significant gaps in research, practical knowledge, and infrastructure. The Tanzanian government recognizes the importance of adopting digital health technologies, including AI and ML, as outlined in the Tanzania National Digital Health Strategy 2019–2021. However, more work is needed to build capacity and develop locally relevant AI-driven solutions for public health.

Given the high burden of climate-sensitive waterborne diseases and the growing impact of climate change on public health, this study aims to assess the knowledge, perceptions, challenges, and opportunities related to using ML in predicting these diseases among public health officers in Tanzania. The study contributes to providing insights for designing AI-based and data-driven approaches to strengthen public health resilience to climate-sensitive waterborne diseases in Tanzania. Specifically, the objectives are:

1. To assess the knowledge of information and communication technology (ICT) and AI/ML among environmental health officers in selected study areas in Tanzania.
2. To evaluate the perceptions of environmental health officers regarding the application of AI/ML in predicting climate-sensitive waterborne diseases.
3. To identify the perceived challenges in integrating AI/ML into public health practices in Tanzania.

The rest of the paper is organized as follows: Section 2 outlines the data collection approach and analysis techniques. The Section 3 presents the key findings, followed by the Section 4, which interprets and compares these results. Finally, the Conclusions summarize key insights and propose future research directions.

## 2 Methods

### 2.1 Study area

The research was conducted across three district councils in Tanzania: Morogoro Municipal Council (MC), Ilala City Council (CC) (in Dar es Salaam), and Dodoma CC. These districts were selected as part of the larger ACHE project (AI for Climate and Health), which aimed to collect a comprehensive dataset on climate-sensitive waterborne diseases to support predictive machine learning models in public health in Tanzania. The ACHE project encompassed five district councils. Of the five councils engaged in the ACHE project, three were included in this study; Temeke was used for piloting the data collection tool, while Singida was excluded due to unavailability of EHOs during the data collection period, as many were on leave.

Morogoro MC is an urban district located in eastern Tanzania, with a population of ~471,000. Ilala District in Dar es Salaam is a highly urbanized and densely populated area, serving as a commercial and administrative hub, with a population exceeding 1.6 million. Dodoma City Council is situated in the central part of the country and serves as the national capital, has a population of around 765,000. These population statistics are according to the National Census report of 2022 [The United Republic of Tanzania (URT) et al., 2024]. Despite variations in their geographic locations and population sizes, EHOs across these districts operate under uniform national policy frameworks. They are recruited based on standardized qualifications in Environmental Health and share similar job descriptions and professional responsibilities across the country.

The survey reported in this paper was conducted concurrently with the ACHE project, not after its completion, meaning that prior experience in the project did not influence participant selection. Tanzania has relatively consistent climate conditions across districts, and the roles and responsibilities of EHOs are the similar nationally. For these reasons, the study findings remain broadly applicable to other Tanzanian districts beyond those included in this sample.

The dataset collected through the ACHE project is openly accessible via the Zenodo repository (Fue et al., 2025), to allow AI/ML researchers and data scientists to use it for training predictive ML models on climate-sensitive waterborne disease outbreaks. A detailed description of the dataset will be presented in a separate paper, while the initial baseline models developed using the data are also covered in another companion paper. This baseline survey focused on assessing the readiness and familiarity of EHOs with AI/ML, a crucial step before deploying ML-supported public health interventions.

### 2.2 Participants

The study focused on Environmental Health Officers (EHOs), for two reasons: their involvement in the projects data collection for open datasets for predictive ML; and their formal and daily duties, which include monitoring and controlling environmental hazards, enforcing public health regulations, conducting disease surveillance, and promoting community health initiatives to prevent and manage diseases. The selected districts comprised a total of 106 wards (as of December 2024), each ideally assigned one EHO. However, due to personnel shortages, some EHOs were responsible for multiple wards, resulting in a total of 76 EHOs available across the three councils. While participation in the study was voluntary, selection bias is unlikely to have been a major concern. The survey targeted all the available 76 EHOs who were actively serving across the three councils.

### 2.3 Sampling method

Due to the specialized expertise needed for this baseline study and the relatively small population size, the study did not employ a sample size calculation in its traditional sense; it aimed to include all the 76 EHOs who were actively on duty at the time of the study. Since this was a small, well-defined population, we aimed to include all EHOs rather than rely on sampling calculations. As such, clustering by study site was not considered relevant for sample size determination. This census-style approach was appropriate for capturing baseline knowledge and perceptions among the full workforce available in the selected areas. Since participation was voluntary in accordance with research ethics, which requires informed consent, only a subset of EHOs ultimately took part in the survey.

### 2.4 Data collection

Data was gathered using a structured questionnaire, which was administered in two formats: a printed version and an online version via Google Forms. The questionnaire was organized into five thematic sections. The first section collected demographic information, mainly sex, age group, and education level. The second section assessed the respondents' familiarity with and use of ICT tools in public health. The third section explored methods EHOs currently use to predict and manage weather-sensitive waterborne diseases. The fourth section assessed their knowledge of AI and ML, along with their percepectives on the applicability of these technologies in public health. The final section explored the perceived challenges of implementing AI and ML in public health in this context. To capture perceptions effectively, the questionnaire used Likert scale questions, ranging between 4-point and 5-point scales depending on the nature of the question.

The survey instrument was developed by the project team based on a thorough literature review and insights from preliminary fieldwork in Morogoro, where researchers are based. Although not adapted from an existing validated tool, the questionnaire underwent pilot testing in Temeke District with nine EHOs. Their feedback was used to refine the language, sequence, and content of several questions, including the addition of items targeting potential causative factors behind responses, particularly regarding experiences and attitudes toward AI/ML. These refinements enhanced relevance of the tool, comprehensiveness, and overall effectiveness in capturing the study's intended objectives. The survey was administered in English, as this is the official language of communication among EHOs in Tanzania.

### 2.5 Data analysis

The collected data was analyzed using both descriptive and inferential statistical methods, with Python scripts. Descriptive statistics were used to summarize demographic information and identify overall trends. For inferential statistics, the Chi-square test was applied to assess associations between nominal and ordinal variables, while Kendall's Tau was utilized to evaluate relationships among ordinal variables. Kendall's Tau was chosen due to the relatively small sample size.

### 2.6 Ethical clearance and research permit

The study adhered to all required ethical guidelines, obtaining official research clearance and permits before data collection commenced. Ethical approval was secured from Sokoine University of Agriculture (Research Clearance No. SUA/DPRTC/R/126/CoNAS/2/2023/3), while government research permits (Permit No. JC.156/254/01) were granted by the President's Office, Regional Administration, and Local Government Authorities (PO-RALG). In addition to these central approvals, further permissions were obtained at the regional and district levels. Specifically, at each region, authorization was sought from the Regional Administrative Secretary's (RAS) office, which formally introduced the research team to the respective district authorities. Following this, each district issued research clearance and introduction letters to the Ward Executive Officers (WEOs) and EHOs, requesting their cooperation and support during data collection.

## 3 Results

### 3.1 Demographics information

Questionnaires were distributed to all the 76 EHOs across the three councils. Of these, 50 were completed and submitted successfully, a response rate of 66%. Table 1 shows that the proportion of female and male EHOs responses to the questionnaire is 48 and 52% respectively. The distribution of respondents across the three councils is further illustrated in Table 2. Morogoro MC represented 50% of the total respondents, Dodoma CC represented 26%, and Ilala CC represented 24%. In Morogoro MC, the demographic breakdown revealed that 52% of respondents were male, while 48% were female. In Dodoma CC, the male population constituted 61%, while the female population accounted for 39%. In Ilala CC, the gender distribution among respondents showed that 33% were male, while 67% were female.

**Table 1:** Demographic characteristics of the respondents by frequency distribution.

**Category**	**Group**	**Count**	**Percentage (%)**
Sex	Female	24	48.0
	Male	26	52.0
Education	Diploma	38	76.0
	Bachelor	9	18.0
	Certificate	3	6.0
Age group	18–30	6	12.0
	31–40	23	46.0
	41–50	11	22.0
	51–60	10	20.0

**Table 2:** Distribution of the respondents' gender per council.

**Council**	**Total count**	**Male%**	**Female%**	**% Distribution of the respondents per council**
Morogoro MC	25	52	48	50
Dodoma CC	13	61	39	26
Ilala CC	12	33	67	24

Additionally, Table 1 shows the largest proportion of the respondents were in the age range of 31–40 (46%) whereas the smallest proportion was in the age range of 18–30 (12%). With regard to their education level, Table 1 shows that 76% of the EHOs had Diploma, 18% had Bachelor degree and 6% had Certificate. The education levels of respondents across the three councils are further elaborated in Table 3. Within the Morogoro CC, the data indicates that 4% of participants had a Certificate, 80% held a Diploma, and 16% attained a Bachelor's degree. In Dodoma CC, the educational qualifications were distributed as follows: 15% of individuals had a Certificate, 70% possessed a Diploma, and 15% attained a Bachelor's degree. Within the Ilala CC, it was observed that none of the participants possessed a Certificate, whereas 75% had attained a Diploma and 25% had completed a Bachelor's degree.

**Table 3:** Distribution of respondents' education levels per council.

**Council**	**Total Count**	**Certificate (%)**	**Diploma (%)**	**Bachelor (%)**
Morogoro MC	25	4	80	16
Dodoma CC	13	15	70	15
Ilala CC	12	0	75	25

### 3.2 The familiarity and usage of ICT tools and facilities among EHOs for public health

**Figure 1:**
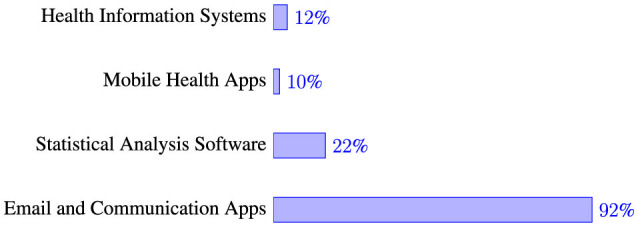
ICT tools usage in public health activities.

When asked to rate their familiarity with information and communication technology (ICT) tools, 46% of participants reported being moderately familiar, 32% felt very familiar (Table 4). Table 4 also shows a smaller proportion of respondents (14%), considered themselves somewhat familiar, while 4% each rated themselves as extremely familiar and not familiar at all. Regarding the frequency of using digital devices (computers, smartphones, tablets) for work-related tasks in the public health sector, Table 4 illustrates that 76% of respondents use these devices daily. Table 4 also shows that 16% use them occassionally in a week, 4% use them at least once a week, and the remaining 4% never use digital devices for public health activities. The study also shows that EHOs in the selected districts employ various ICT tools in public health initiatives. As presented in Figure 1, email and communication applications, such as WhatsApp, accounted for the highest usage at 92%. Statistical analysis software was used by 22% of respondents, health information systems by 12%, and mobile health applications by 10%.

**Table 4:** ICT familiarity and digital tools usage among EHOs by frequency distribution.

**Category**	**Group**	**Count**	**Percentage (%)**
Rate your familiarity with using information and communication technology (ICT) tools	Extremely familiar	2	4.0
	Very familiar	16	32.0
	Moderately familiar	23	46.0
	Somewhat familiar	7	14.0
	Not familiar at all	2	4.0
How frequently do you use digital devices (computers, smartphones, tablets) for work-related tasks in the public health sector?	Daily	38	76.0
	Sometimes	8	16.0
	At least once in a week	2	4.0
	Never	2	4.0

### 3.3 Knowledge of AI and ML among EHOs

Table 5 reveals that 54% of the respondents had encountered the terms AI and ML before participating in the study, while 46% had not. On the other hand, Table 5 indicates that 64% were somewhat familiar with the concept of Artificial Intelligence, a notable 30% were not familiar at all, and only 6% were very familiar with the AI concept.

**Table 5:** AI/ML familiarity among EHOs by frequency distribution.

**Category**	**Group**	**Count**	**Percentage**
Had you encountered the term “Machine Learning (ML) or Artificial Intelligence (AI)” before this survey?	Yes	27	54.0
	No	23	46.0
How familiar are you with the concept of Artificial Intelligence (such as Machine Learning)?	Somewhat familiar	32	64.0
	Not familiar	15	30.0
	Very familiar	3	6.0

### 3.4 EHOs observations on waterborne disease trends

**Figure 2:**
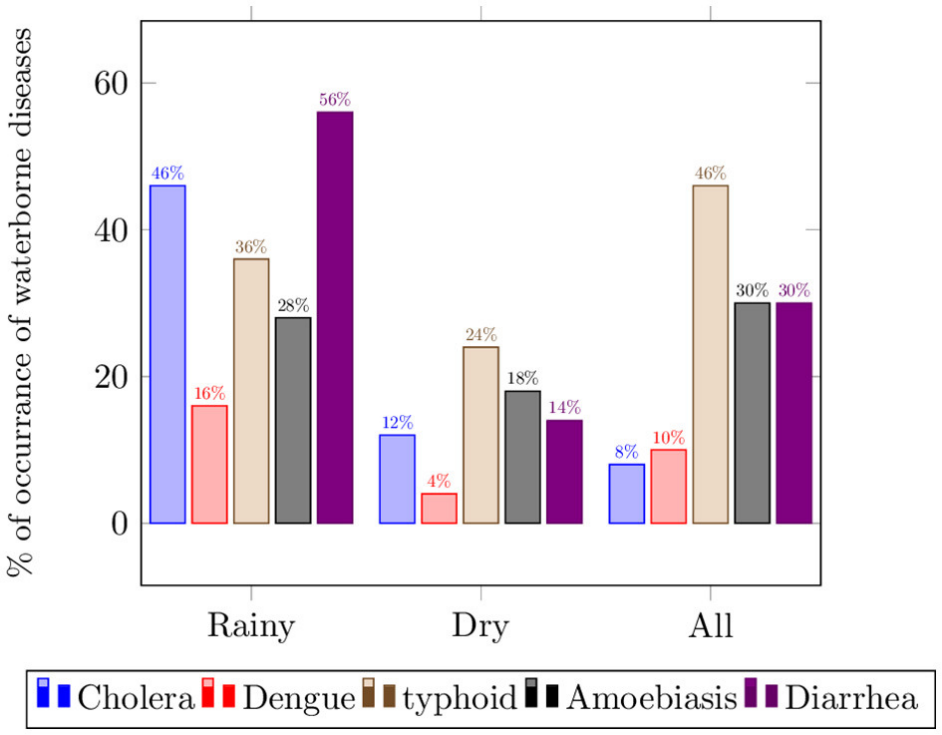
Perceived prevalence of waterborne diseases.

To understand the relationship between weather-sensitive waterborne diseases and weather conditions, the EHOs were asked to identify prevalent waterborne diseases in their area and the associated weather patterns. Figure 2 shows that diarrhea and cholera are reported to be frequently observed during the wet/rainy seasons, with cholera (56%) having more peak occurrence, followed by diarrhea (46%). Cholera was reported to have a substantial decrease in occurrences during the dry season. Typhoid fever, categorized as a seasonal disease, has been reported in both wet (24%) and dry (46%) seasons, but it exhibits higher prevalence during the dry season.

### 3.5 Key data sources for monitoring and predicting WSWB diseases

**Figure 3:**
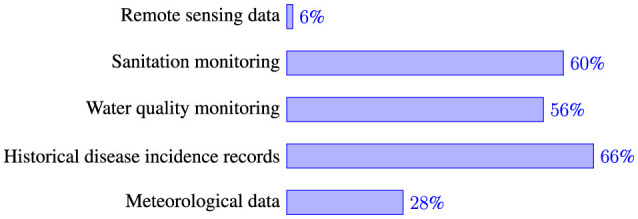
Key data sources for monitoring and prediction of waterborne diseases.

Understanding the data sources used by health professionals is essential for designing effective monitoring systems for weather-related health risks. In this study, respondents were asked to identify the primary data sources they use for tracking and predicting waterborne disease outbreaks in the areas of their jurisdictions in Tanzania. The findings reveal that historical disease incidence records are the most frequently used source, with 66% of respondents depending on them for monitoring and predicting weather-sensitive waterborne (WSWB) disease outbreaks (Figure 3). These records are primarily obtained from the DHIS2, a national health data reporting and monitoring system, as well as news reports on disease outbreaks. Additionally, 60% of respondents reported using sanitation data, while 56% relied on water quality monitoring data. Meteorological data are utilized by 14% of the respondents who reported relying on them for predicting WSWB diseases. However, despite the valuable environmental insights offered by remote sensing data, only 6% of respondents reported using this source.

### 3.6 The potential of integrating AI and ML into existing WSWB disease prediction and management strategies

Regarding perceptions of AI/ML in enhancing the accuracy and timeliness of disease outbreak predictions amid changing climate patterns, 50% of respondents agreed, while 20% strongly agreed. Meanwhile, 26% were uncertain, and 4% disagreed, as shown in Table 6. When asked whether AI/ML could replace or complement traditional methods for predicting and managing waterborne diseases, the same Table 6 reveals that 40% believed AI/ML could serve as a full replacement. Additionally, 26% felt it could fully complement traditional methods, 22% believed it would provide partial support, and 12% thought it would not replace traditional approaches at all.

**Table 6:** Perceptions of EHOs on AI/ML by frequency distribution.

**Category**	**Group**	**Count**	**Percentage**
ML/AI contributes to more accurate disease outbreak predictions	Strongly agree	10	20.0
	Agree	25	50.0
	No idea	13	26.0
	Disagree	2	4.0
	Strongly disagree	0	0.0
Can ML/AI replace or complement traditional methods?	Replace	20	40.0
	Fully complement	13	26.0
	Partially complement	11	22.0
	Not at all	6	12.0
Confidence in AI/ML for waterborne disease management	Very confident	9	18.0
	Confident	29	58.0
	Not sure	12	24.0
	Not confident	0	0.0
Healthcare professionals' trust in AI/ML for disease prediction	Strongly agree	10	20.0
	Agree	27	54.0
	No idea	12	24.0
	Disagree	1	2.0
	Strongly disagree	0	0.0

Furthermore, Table 6 indicates that 58% of respondents expressed confidence in the reliability and accuracy of machine learning models for predicting waterborne diseases, while 18% were very confident, and 24% remained uncertain. In terms of healthcare professionals' trust in machine learning algorithms for disease prediction and management, 54% of respondents agreed, 20% strongly agreed, 24% were unsure, and 2% disagreed (Table 6).

**Figure 4:**
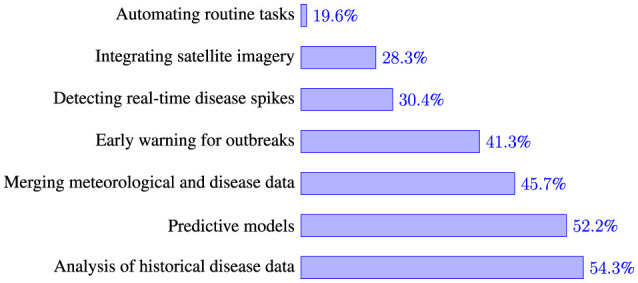
Different ways of integrating AI/ML in waterborne diseases prediction and management.

EHOs identified multiple ways in which AI/ML could be integrated into existing disease prediction and management strategies (Figure 4). The most commonly selected approach, chosen by 54.3% of respondents, was utilizing AI/ML to analyze historical disease data. Similarly, 52.2% envisioned developing predictive models to enhance outbreak forecasting. Around 45.7% suggested integrating meteorological data, water quality data, and disease incidence records to provide a more comprehensive understanding of disease trends. Additionally, 41.3% saw AI-driven early warning systems as a valuable tool for proactive disease management. Approximately 30.4% supported using AI to detect sudden spikes in disease incidence in real time by analyzing data streams from various sources. Meanwhile, 28.3% highlighted the potential of incorporating satellite imagery with disease data to assess the relationship between land cover changes and outbreaks. Lastly, 19.6% envisioned AI automating routine tasks to improve efficiency in disease monitoring and response efforts.

### 3.7 Demographic factors and their influence on AI perceptions and ICT usage

Table 7 explores how respondents' gender relates to various factors, including familiarity with ICT (ict_familiarity), usage of digital tools in public health (ict_usage), prior exposure to AI/ML terms (ai_ml_encounter), familiarity with AI (ai_familiarity), perceptions of AI/ML in predicting weather-sensitive waterborne diseases (ai_ml_prediction), belief in AI replacing traditional methods (ai_replace_traditional), and trust in ML decisions (ml_trust). Among these associations, only the relationships between sex and both prior exposure to AI/ML terms and AI familiarity were statistically significant (*p* = 0.049) and (*p* = 0.006), respectively. On the other hand, Table 8 examines whether age group (age_group) is associated with the same set of variables. The results indicate no statistically significant relationships between age and any of the tested factors. The low correlation coefficients suggest minimal association, while the high p-values indicate that any observed trends are likely due to chance. Since none of the associations reach statistical significance (*p* < 0.05), we cannot conclude that age influences AI/ML awareness, ICT familiarity and usage, or related perceptions in a meaningful way.

**Table 7:** Relationship between sex and other variables.

**Variable 1**	**Variable 2**	**Statistic (*X*^2^)**	***p*-value**
Sex	ICT familiarity	2.708	0.608
	ICT usage	2.872	0.412
	AI/ML encounter	3.862	0.049
	AI familiarity	10.337	0.006
	AI/ML prediction	5.566	0.135
	AI replaces traditional	3.688	0.297
	ML trust	3.474	0.324

**Table 8:** Relationship between age group and other variables.

**Variable 1**	**Variable 2**	**Kendall's *T***	***p*-value**
Age group	ICT familiarity	0.055	0.659
	ICT usage	0.049	0.701
	AI/ML encounter	–0.100	0.450
	AI familiarity	0.045	0.726
	AI/ML prediction	–0.200	0.107
	AI replaces traditional	–0.182	0.135
	ML trust	–0.070	0.574

Table 9 investigates how education level (education) correlates with ICT and AI-related variables. The findings reveal that education level is significantly associated with the use of ICT tools in public health (*p* = 0.012), indicating that individuals with higher education levels are more likely to use such tools. However, all other relationships show weak or negligible correlations and are not statistically significant.

**Table 9:** Relationship between education and other variables.

**Variable 1**	**Variable 2**	**Kendall's *T***	***p*-value**
Education	ICT familiarity	0.144	0.271
	ICT usage	0.341	0.012
	AI/ML encounter	–0.027	0.845
	AI familiarity	0.029	0.831
	AI/ML prediction	0.138	0.293
	AI replaces traditional	0.112	0.387
	ML trust	0.128	0.332

### 3.8 Challenges and opportunities for AI and ML adoption in public health

**Figure 5:**
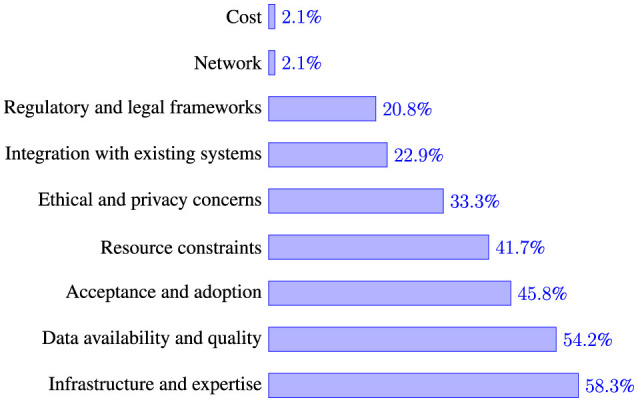
Challenges for integration of AI/ML in waterborne disease prediction and management.

To evaluate the challenges and opportunities associated with integrating machine learning (ML) into Tanzania's public health sector, respondents highlighted several key barriers (see Figure 5). The most pressing challenge, cited by 58.3% of the participants, was the lack of technical infrastructure and expertise necessary for effective implementation. Data availability and quality were also major concerns, with 54.2% identifying these as critical obstacles.

Other notable challenges include the acceptance and adoption of AI/ML technologies (45.8%) and resource constraints, such as funding and skilled personnel shortages (41.7%). Ethical and privacy concerns were raised by 33.3% of the respondents, while 22.9% pointed to difficulties in integrating AI/ML with existing public health systems. Regulatory and legal frameworks were also seen as potential barriers, with 20.8% recognizing the need for clearer guidelines and policies.

Although less frequently mentioned, network connectivity issues and financial costs were identified as potential hindrances by a small percentage (2.1%) of respondents. These findings highlight both the technical and socio-economic challenges that must be addressed to fully leverage AI/ML for public health improvements in Tanzania.

## 4 Discussion

The findings of this study offer important reflections on the feasibility of integrating machine learning (ML) into public health practices for managing weather-sensitive waterborne diseases (WSWDs) in Tanzania. The discussion focuses on the key thematic areas of ICT and AI/ML knowledge among environmental health officers (EHOs), their perceptions of AI/ML applications, and the critical challenges and opportunities that influence the adoption of these technologies.

### 4.1 Knowledge of ICT and AI/ML among EHOs

The study indicates that while EHOs have a reasonable level of familiarity with general ICT tools, their understanding of AI and ML remains limited. This knowledge AI and ML gap is a potential barrier to the successful adoption of AI-driven solutions in public health. The findings suggest that despite a moderate engagement with digital tools in their daily activities, many EHOs have not had significant exposure to AI/ML concepts, which may limit their ability to evaluate and implement such technologies effectively. One approach to bridging this gap is targeted training programs and continuous professional development opportunities to equip EHOs with the necessary competencies in AI/ML applications.

Targeted training programs have proven effective in enhancing digital competencies among health professionals, thereby facilitating the adoption of AI and ML technologies in public health. For instance, the Sustainable Healthcare with Digital Health Data Competence (Susa) project in Europe, funded by the EU's Digital Europe programme, aims to equip over 6,500 health professionals with advanced digital skills, including AI integration, through structured bachelor's and master's programs, as well as lifelong learning modules. This initiative illustrates the importance of involving healthcare workers early in the technology development process to ensure successful implementation (Cookson, 2025). Similarly, in African contexts, studies have highlighted that AI can significantly improve public health surveillance and disease monitoring. However, the lack of skilled health professionals remains a barrier, emphasizing the need for targeted training to harness AI's full potential (Tshimula et al., 2024).

### 4.2 Utilization of key data sources for efficient monitoring and prediction of WSWB diseases

EHOs rely on traditional methods to predict waterborne diseases, primarily through manual reviews of historical disease outbreak records and observable weather patterns, without the support of dedicated tools or systems. This approach limits the effective utilization of available data. EHOs specifically reported underutilization of key sources such as meteorological and remote sensing data. Lack of integrated systems or analytical tools that can process and interpret such data effectively, hindering the potential for more accurate and timely disease prediction. Meteorological data such as precipitation, temperature, and humidity are essential for identifying environmental variables that affect disease outbreaks (Chowdhury et al., 2018). Likewise, remote sensing data offers significant insights into changes in land cover, variations in water quality, and environmental risk factors, which are essential for early warning systems (Mashala et al., 2023; Deng et al., 2024). The limited utilization of these data sources could be due to a lack of tools or systems that utilize these data, which hinders the ccuracy of disease prediction and hinders the effectiveness of proactive interventions. Strengthening capacity-building initiatives is essential to promote the adoption of advanced analytical methods, such as ML algorithms, enabling the effective integration of diverse data types for comprehensive analysis and more accurate predictive modeling.

### 4.3 Perceptions of AI/ML in managing weather-sensitive waterborne diseases

EHOs generally express optimism about the role of AI and ML in enhancing disease prediction and management. However, this optimism is accompanied by uncertainties, particularly regarding the efficacy of AI/ML models in real-world settings. While some respondents view AI/ML as a potential replacement for traditional methods, others see it as a complementary tool that should be integrated cautiously. This divergence in perception underscores the need for clear implementation frameworks and capacity-building initiatives that illustrate how AI/ML can augment rather than replace existing public health strategies. Ensuring that these technologies are positioned as decision-support tools rather than automated replacements for human expertise will be crucial for fostering trust and facilitating adoption. This divergence in perception is not unique to our study area; similar sentiments have also been reported across low- and middle-income countries.

There are significant advancements in AI-driven health initiatives within low- and middle-income countries, with African nations such as Kenya, Nigeria, and Ghana leading the way. Nonetheless, most of these AI systems have remained confined to academic environments, mainly concentrating on model training, validation, and testing, without advancing to practical implementation (Ndagire et al., 2022; Baik et al., 2020). This underscores a notable disparity between the advancement of AI technologies and their limited uptake in the healthcare sector. The slow uptake of AI and other new technologies in healthcare, as expressed in literature, is largely due to varying perceptions among stakeholders. Some healthcare professionals worry that technology could threaten their independence, patient care, or be used as a means of administrative control. However, some view it as a means to improve patient engagement. Patients generally embrace technology because it gives them more choice in treatment (Safi et al., 2018; Mirugwe, 2024). Effective change management is crucial for technology adoption since insufficient inclusive engagement in technology creation sometimes causes resistance.

### 4.4 Gender and AI awareness

Among the various variables examined in relation to gender, only two, prior exposure to AI/ML terminology and self-reported familiarity with AI, demonstrated statistically significant associations. These findings suggest that gender plays a notable role in shaping awareness and engagement with artificial intelligence and machine learning. Specifically, one gender group appears more likely to have encountered or interacted with AI/ML concepts, which may reflect differences in access to relevant educational resources, workplace roles, or broader socio-cultural dynamics that influence exposure to digital technologies.

This observation aligns with existing literature highlighting persistent gender gaps in digital literacy and attitudes toward AI. For instance, Russo et al. (2025) reported that women tend to exhibit greater anxiety toward AI, hold less favorable attitudes, engage with the technology less frequently, and perceive themselves as having lower knowledge compared to men. These patterns are reflected in our sample, where female respondents reported lower familiarity and exposure. Moreover, as noted by Armutat et al. (2024), structural barriers, including gender stereotypes, unequal access to educational opportunities, and systemic discrimination, continue to limit women's participation in AI and other technology-driven fields.

The implications of these findings are significant for efforts to promote inclusive AI literacy and workforce development. Addressing gender disparities in AI knowledge and exposure requires targeted interventions, such as gender-sensitive training programs, outreach initiatives in underrepresented communities, and policies aimed at dismantling structural barriers in education and employment. Without deliberate efforts to close these gaps, the underrepresentation of women in AI-related domains may persist, ultimately limiting the diversity of perspectives and innovations in this rapidly evolving field.

### 4.5 Challenges and opportunities

Despite the potential of AI and ML, several significant challenges hinder their adoption in Tanzania. These challenges, if unaddressed, could limit the applicability, scalability, and sustainability of AI-driven public health interventions in Tanzania.

One of the most pressing challenges is the lack of infrastructure. Deploying AI/ML models effectively requires robust digital infrastructure, including reliable internet access, sufficient computational power, and a stable electricity supply—resources that remain insufficient in many parts of Tanzania. The absence of these foundational elements restricts the ability to run complex AI models in real time and limits opportunities for cloud-based computing solutions. Without substantial investments in digital infrastructure, AI/ML applications risk remaining confined to research settings without meaningful real-world impact. This also limits affect the deployment of Electronic Health Record Systems (EHRS) in Tanzanian primary healthcare facilities, according to Mwogosi (2024). Furthermore, the limited availability of AI/ML specialists in the public health sector means that even where infrastructure is available, there are few personnel with the necessary skills to develop, implement, and maintain AI-driven solutions. Addressing this issue requires incorporating AI/ML education into public health curricula and establishing continuous professional training programs tailored for EHOs and other key stakeholders. The second issue is data availability and quality, which also present significant barriers. Many public health decisions rely on historical disease records, which are often incomplete, inconsistent, or outdated. Additionally, the integration of diverse data sources—such as meteorological data, water quality indicators, and satellite imagery—remains underutilized. The absence of standardized data collection protocols and insufficient data-sharing mechanisms among institutions further exacerbate the challenge. Without high-quality, well-structured datasets with supporting infrastructure in place AI/ML models cannot be effectively trained to generate reliable predictions.

According to Yonazi (2012) and Muhunzi et al. (2024), poor data governance and insufficient investment in digital systems infrastructures hinder the use of big data analytics in healthcare in many developing nations. However, high-income countries have strong digital infrastructures to facilitate AI and machine learning in healthcare. Electronic health record systems are widely used in developed countries due to its standardization, interoperability, and immediate data availability (Slawomirski et al., 2023). The European Health Data Space's open data regulations and platforms have improved data accessibility and innovation (Hussein et al., 2023). The disparities underscore a notable digital divide, with high-income nations more equipped to utilize data-driven health solutions, whereas developing nations face the challenge of addressing essential infrastructure and data quality issues first. Despite these challenges, Arinze (2024) suggests that East African Open Data Initiatives may pave the way for more advanced digital health applications. Strengthening data governance frameworks and fostering collaborations between public health agencies and research organizations could improve data accessibility and the development of data-driven solutions in the health sector.

Beyond technical and data-related constraints, there are significant sociocultural and institutional challenges related to the acceptance and adoption of AI/ML in public health. Resistance to new technologies is common when professionals perceive them as disruptive or when their benefits are not clearly demonstrated. In many cases, EHOs may be skeptical of AI/ML-driven decision-making, particularly if these technologies are introduced without adequate stakeholder engagement. Ethical concerns, such as transparency in AI-driven decision-making and the risk of algorithmic biases, also contribute to hesitancy. To overcome these barriers, there is a need for structured implementation strategies that involve EHOs in the co-design of AI/ML systems, ensuring that these technologies align with their workflows and address real-world challenges in disease management. Therefore, a collective effort is needed to advance research on AI/ML applications for predicting and managing diseases such as WSWD, particularly in low- and middle-income countries (LMICs) such as Tanzania. Research should focus on collecting high quality data and developing AI/ML models that are optimized for resource-constrained environments, ensuring that these technologies are both accessible and applicable. Additionally, studies exploring strategies for capacity-building, infrastructure development, and ethical AI adoption will be essential for facilitating the successful integration of AI-driven solutions in public health.

### 4.6 Limitations of the study

While the findings of this study provide valuable insights into the readness and perceptions of EHOs ragrding AI/ML in public health, seral limitations must be acknowledged. First, the sample consisted of EHOs who participated in a specific data collection project (ACHE), which may introduce selection bias. Although their participation was not based on prior experience with AI/ML, their engagement in a broader health data initiative may reflect a more proactive or accessible segment of EHOs, potentially limiting generalizability to all EHOs across Tanzania.

Second, the survey instrument, while piloted and refined through feedback from EHOs in Temeke district, was not adapted from pre-existing validated tools. Although this iterative process improved clarity and relevance, the absence of a formally validated instrument introduces a degree of measurement bias.

Third, the survey was administered in Kiswahili, the national language, to ensure accessibility and reduce language-related misunderstandings. However, minor interpretation differences across regions may have influenced how some questions were perceived or answered.

Lastly, the study relied on self-reported data, which is subject to recall and social desirability biases. Future research should aim to expand the sample across more diverse districts and incorporate mixed-methods approaches—such as in-depth interviews and focus group discussions—to enrich and triangulate the findings.

## 5 Conclusion and future work

This study explored the knowledge, perceptions, and preparedness of Environmental Health Officers (EHOs) in selected Tanzanian districts regarding the application of machine learning (ML) in predicting weather-sensitive WSWDs. The findings reveal that while general ICT familiarity among EHOs is reasonably strong, there remains a substantial gap in awareness and understanding of AI and ML concepts. This gap presents a critical challenge for integrating ML-based systems into public health decision-making processes.

Importantly, the study highlights several perceived barriers to ML adoption from the perspective of frontline health workers, including limited technical skills, data quality concerns, and institutional readiness. While the study did not directly assess existing infrastructure or data systems, the perceptions gathered from EHOs offer valuable insights into the enabling conditions required for future ML applications.

Rather than presenting ML as an immediately deployable solution, this study emphasizes the importance of targeted capacity-building and policy support to prepare the public health workforce for digital transformation. Future research should complement these findings by examining actual data readiness, infrastructure capabilities, and the development of contextually appropriate ML models for low-resource settings. Pilot interventions, co-designed with health professionals, may also help bridge the gap between potential and practical implementation of ML for disease prediction and control.

## Data Availability

All relevant data are included within the article and its supplementary materials. Further inquiries regarding the generated and analyzed data from this study can be obtained from the corresponding author upon request.
