# Supplementary material for: Assessing the potential for application of machine learning in predicting weather-sensitive waterborne diseases in selected districts of Tanzania
Source: Front Artif Intell. 2025 Jun 4;8:1597727. doi: 10.3389/frai.2025.1597727 (PMC12174062; doi:10.3389/frai.2025.1597727)
Supplement: Supplementary file 1 [file Data_Sheet_1.pdf]

QUESTIONNAIRE FOR HEALTH OFFICERS

Title: Assessment of ICT Literacy and the Understanding of Potential Applications of Machine Learning in Public Health for Climate Sensitive Waterborne Disease Management in Developing Countries: A case of Tanzania

Introduction: This questionnaire is designed to assess the knowledge and understanding of public health officers regarding ICT tools especially on machine learning applications in addressing waterborne diseases in developing countries, with a specific focus on Tanzania. The objective is to gather insights into the understanding of waterborne diseases and their awareness of ICT such as ML technologies in the context of public health.

* Indicates required question

Section 1: Demographic Information

1. What is our age group? *

   Mark only one oval.

   - 18-30
   - 31-40
   - 41-50
   - 51-60

2. What is your sex? *

   Mark only one oval.

   - Male
   - Female

3. Educational Level *

Check all that apply.

- [ ] Secondary School
- [ ] Certificate
- [ ] Diploma
- [ ] Bachelor Degree
- [ ] Postgraduate (Masters/PhD)
- [ ] Other: ______________________

4. 4. Designation *

____

5. 5. Duty station(s) *

____

Section 2: ICT Literacy in the public health sector in Tanzania

6. Please rate your familiarity with using information and communication technology (ICT) tools

Mark only one oval.

- ( ) Not Familiar at All
- ( ) Somewhat Familiar
- ( ) Moderately Familiar
- ( ) Very Familiar
- ( ) Extremely Familiar

7. How frequently do you use digital devices (computers, smartphones, tablets) for work-related tasks in the public health sector?

Mark only one oval.

- Never
- Sometimes
- At least once in a week
- Daily

8. Which ICT tools do you currently use for public health related activities? (Select all that apply)

Check all that apply.

- [ ] Email and Communication Apps (e.g., WhatsApp)
- [ ] Statistical Analysis Software (e.g., MS Excel, SPSS, R, Python)
- [ ] Health Information Systems (HIS)
- [ ] Mobile Health (mHealth) Apps
- [ ] Other: ______________________

9. Have you received any formal training or workshops on information and communication technology (ICT) in the context of public health?

Mark only one oval.

- Yes
- No

Section: Knowledge and current practices for predicting and managing of waterborne diseases

10. Please select climate-sensitive waterborne diseases that are common in your area and their associated weather conditions

Check all that apply.

	Cholera	Dengue Fever	Typhoid Fever	Amoebiasis	Diarrhea
**Rainy season**	☐	☐	☐	☐	☐
**Dry season**	☐	☐	☐	☐	☐
**All weather**	☐	☐	☐	☐	☐

11. Choose other environmental factors (other than climatic conditions) in your area that may be contributing to waterborne diseases: (Select all that apply)

Check all that apply.

- ☐ Poor Hygiene Practices
- ☐ Population Density
- ☐ Poorly maintained water treatment
- ☐ Poor Waste Management
- ☐ Poor sanitation
- ☐ Poor drainage system
- ☐ Other: ______________________

12. What are the primary data sources you rely on for monitoring and predicting climate-related trends in waterborne disease outbreaks in Tanzania? (Select all that apply)

Check all that apply.

- ☐ Meteorological data
- ☐ Historical disease incidence records
- ☐ Water quality monitoring
- ☐ Sanitation monitoring
- ☐ Remote sensing data (e.g., satellite imagery)
- ☐ Other: ______________________

Section 4: Knowledge and perception of machine learning applications

13. Had you encountered the term "Machine Learning (ML) or Artificial Intelligence (AI)" before participating in this survey?

Mark only one oval.

- Yes
- No

14. How familiar are you with the concept of Artificial Intelligence (such as Machine Learning)?

Mark only one oval.

- Not Familiar
- Somewhat Familiar
- Very Familiar

15. Machine Learning or Artificial Intelligence can contribute to more accurate and timely predictions of disease outbreaks under changing climate patterns

Mark only one oval.

- Strongly agree
- Agree
- No idea
- Disagree
- Strongly Disagree

16. Can Machine Learning or AI replace or complement traditional methods in the prediction and management of waterborne diseases?

Mark only one oval.

- Replace
- Fully complement
- Partially complement
- Not at all

17. What ways do you envision in which machine learning or AI could be integrated into existing disease prediction and management strategies? (Select all that apply)

Check all that apply.

- [ ] Analysis of historical disease data
- [ ] Developing Predictive models
- [ ] Merging meteorological data, water quality data, and disease incidence records to provide a holistic view of disease trends
- [ ] Integrating satellite imagery with disease data and correlating land cover changes and disease outbreaks
- [ ] Detecting sudden spikes in disease incidence in real time using data streams from various sources
- [ ] Providing early warning for potential outbreaks
- [ ] Automating routine tasks
- [ ] Other: ____________________

18. How confident are you in the reliability and accuracy of predictions made by machine learning models in the context of waterborne disease management?

Mark only one oval.

- Very confident
- Confident
- Not sure
- Not Confident

19. Healthcare professionals would trust the decisions made by machine learning algorithms when it comes to disease prediction and management.

Mark only one oval.

- Strongly agree
- Agree
- No idea
- Disagree
- Strongly disagree

Section 5: Challenges and Considerations

20. What challenges, if an, do you foresee when integrating machine learning into public health practices in Tanzania? (Select all that apply)

Check all that apply.

- [ ] Data availability and quality
- [ ] Technical infrastructure and expertise
- [ ] Ethical and privacy concerns
- [ ] Resource constraints
- [ ] Acceptance and adoption
- [ ] Regulatory and legal frameworks
- [ ] Integration with existing systems
- [ ] Other: ______________________

Thank you

Thank you for participating in this questionnaire. Your expertise and insights are invaluable in enhancing the understanding of Machine Learning applications in Public Health, specifically in the context of climate-sensitive waterborne diseases.

This content is neither created nor endorsed by Google.

Google Forms
